# Estimating influenza A subtype ratios among critical care admissions in England

**DOI:** 10.1017/S0950268825100812

**Published:** 2026-01-12

**Authors:** Hannah Sophie Wolmuth-Gordon, Suzanne Elgohari, Gavin Dabrera, Rebecca E. Green

**Affiliations:** Respiratory Viruses, Immunisations and Vaccine-Preventable Diseases, UK Health Security Agency – Colindale, UK

**Keywords:** Influenza, respiratory virus, subtype, transmission, hospitalised, seasonal

## Abstract

We analysed weekly influenza A intensive care unit (ICU) or high dependency unit (HDU) admissions reported by age group and subtype by NHS trusts in England through mandatory surveillance during the 2023–2024 influenza season. We investigated whether subtype reporting varied with patient age group, NHS trust type and region. We estimated the subtype ratio and explored whether this estimate varied among subsets of trusts grouped by the regularity of subtype reporting. Our aim was to explore factors relating to subtype reporting and investigate how these affect subtype ratio estimates. 112 NHS trusts reported data, with 86 trusts reporting influenza A cases and 28 trusts reporting subtyped influenza A cases. The proportion of subtype reporting trusts varied with region and trust type, but not patient age group. The estimated ratio of influenza A(H1N1)pdm09 to influenza A(H3N2) was 3.13 (95% CI: 2.17, 4.51), indicating that influenza A(H1N1)pdm09 was dominant; this was approximately similar across levels of regularity of trust subtype reporting. The accuracy of subtype ratio estimates depends on the availability of influenza A subtype information and data representativeness. We identified low levels of subtype reporting, which likely limits early recognition of new influenza strains and informing of the prescription of antivirals in influenza outbreaks.

## Introduction

Influenza is an infection of the respiratory tract that peaks during the winter in temperate climates and can cause severe disease [1]. Influenza in humans is caused by influenza A, B or C type viruses, with most human illness caused by influenza A and B. Influenza A causes seasonal epidemics the most frequently and multiple subtypes of the influenza A virus typically circulate simultaneously. Currently, in England, influenza A(H1N1)pdm09 and influenza A(H3N2) are in circulation [2]. Understanding the distribution of influenza A subtypes in circulation is necessary to inform patient treatment, such as antiviral choice [3], and can help inform the strain composition of the influenza vaccines for the following season [4]. Admission into intensive care units (ICUs) or high dependency units (HDUs) is an established indicator of severe disease for influenza surveillance in England and a recommended indicator by the World Health Organisation and the European Centre for Disease Prevention and Control [5]. This surveillance enables the monitoring of admission counts and the proportions of these cases attributed to different influenza types and subtypes. Furthermore, the inclusion of unsubtypable influenza A cases in this surveillance supports the identification of emerging non-seasonal subtypes prior to the widespread use of specific assays. However, accurately estimating the ratio of influenza subtypes circulating in a population is challenging, since not all influenza A cases are reported with subtype information [6]. Consequently, influenza subtype data may not be representative of cases across the whole population.

Here, we analyse influenza ICU–HDU admissions data from a surveillance system that monitors severe acute respiratory infection admissions into secondary care in England. The surveillance of influenza ICU–HDU admissions is mandatory for all acute NHS trusts in England [7]. We analysed weekly influenza A cases admitted to ICU–HDU reported by NHS trusts in England via this surveillance system during the winter influenza season 2023–2024. We described the proportion of cases reported with subtype information. We investigated whether subtype reporting varied across factors such as patient age group, trust type and region. In addition, we estimated the subtype ratio based on this data and explored whether this estimate varied according to how regularly trusts report subtyped cases. Our primary aim was to explore factors relating to subtype reporting and investigate how these factors might affect subtype ratio estimates, rather than precisely calculating influenza A subtype ratios.

We analysed weekly counts of new influenza ICU or HDU admissions submitted by trusts via the surveillance system in England during the winter influenza season 2023–2024 (week beginning 2/10/23 to 13/5/2024 inclusive). Each week, NHS trusts report the number of new influenza ICU or HDU admissions in each age category (<6 months, 6–11 months, 1, 2, 3, 4, 5, 6, 7, 8, 9, 10, 11, 12, 13, 14, 15, 16, 17–44, 45–49, 50–54, 55–64, 65–74, 75–84, 85+ years) and the type/subtype of influenza to the UK Health Security Agency (UKHSA) surveillance system. Age groups are combined into wider groups (<1, 1–4, 5–14, 15–44, 45–54, 55–64, 65–74, 75–84, 85+ years) for the UKHSA National Flu and Covid surveillance weekly report [8]. Influenza type/subtype is reported as influenza A(H1N1)pdm09, influenza A(H3N2), influenza A(subtype unknown) (if an assay was used that does not distinguish between influenza A subtypes or information is not available), influenza A unsubtypeable (if subtyping was conducted and a seasonal subtype was not detected) or influenza B. Cases are defined as any symptomatic individual admitted to ICU–HDU with a test confirmed influenza infection, subject to local trust testing procedure. For this analysis, influenza A unsubtypeable cases were removed from the dataset. Analyses were conducted in R version 4.4.2 ‘Pile of Leaves.’

In the 2023–2024 influenza season, 112 trusts participated in the influenza ICU–HDU admission surveillance. Then, 86 NHS trusts reported 930 influenza A or B admissions to ICU–HDU in England. All 86 trusts with influenza admissions reported influenza A cases and 32.6% (28/86) of trusts reported the subtype of at least one case of influenza A. Overall, there were 119 influenza A(H1N1)pdm09 cases, 38 influenza A(H3N2) cases, 718 A(subtype unknown) cases and 55 influenza B cases reported. Therefore, 17.9% (157/875) of influenza A cases were reported with subtype information.

**Figure 1.**
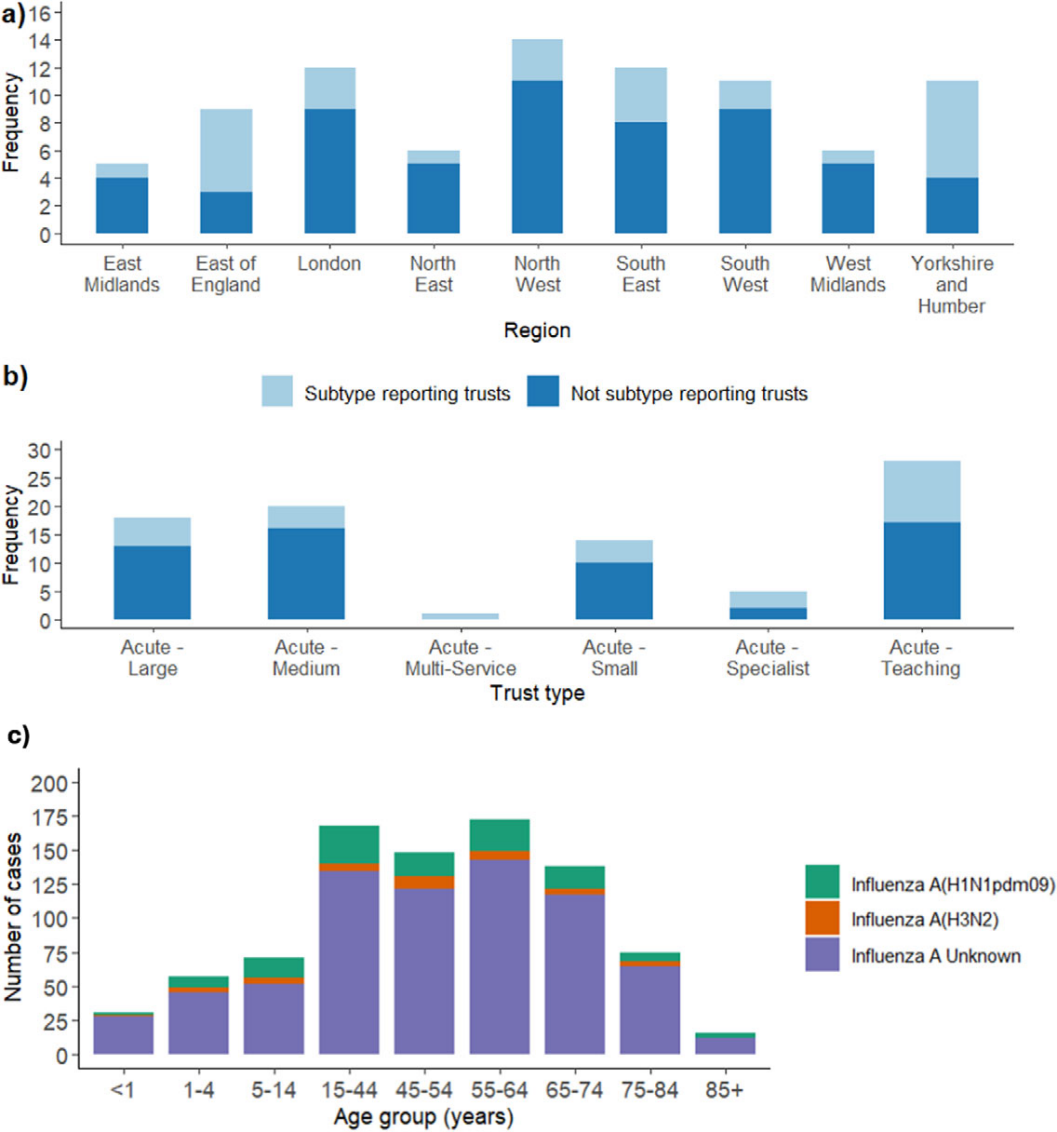
(a) Frequency of trusts that did and did not report subtype for influenza A cases in admissions to ICU–HDU during the influenza 2023–2024 season across regions in England using UKHSA regions. Trusts not reporting influenza A cases during this time period have been removed. (b) same as (a), across NHS trust types. (c) Number of cases of influenza A(H1N1)pdm09, influenza A(H3N2) and influenza A(subtype unknown) reported across age groups in the time period. Data from all NHS trusts included. Influenza B cases have been removed.

NHS trusts and regions in England may have differing capacities to subtype influenza cases, or different testing processes related to clinical pathways for admitted influenza cases. Consequently, we investigated whether the proportion of subtyped influenza cases reported varied by region in England and NHS trust type. NHS trusts were categorized as ‘subtype reporting’ if they reported at least one subtyped case of influenza A in the time period. All other trusts were categorized as ‘not subtyping reporting’. Across all regions in England, there were subtype reporting trusts, but the proportion varied between regions, with the highest in the Yorkshire and Humber region (Figure 1a). Similarly, there were subtype reporting trusts across all NHS trust types with the proportion of subtype reporting trusts varying substantially across trust types (Figure 1b).

Within subtype reporting trusts, we calculated the proportion of weeks that trusts reported a subtyped case of influenza A out of the number of weeks in which any influenza A cases were reported during the 2023–2024 influenza season. Nationally, there were 28 subtype reporting trusts (32.6% of all trusts with at least one reported influenza A ICU–HDU admission). Within the 28 subtype reporting trusts, 18 trusts reported a subtyped case in at least 50% of weeks they reported influenza A cases, of which 13 reported a subtyped case in 80% or more of weeks they reported influenza A cases. To investigate whether the inclusion of trusts that regularly report influenza A subtype affected subtype ratio estimates we calculated subtype ratios using all data, and then restricted the data to the subset of trusts that reported influenza A subtype in at least 50% and then at least 80% of weeks (Table 1). Despite ratio estimates slightly differing, the confidence intervals were large (Table 1) and the three ratio estimates resulted in the same overarching conclusions. All three estimates indicate that influenza A(H1N1)pdm09 and influenza A(H3N2) were both in circulation, with cases of influenza A(H1N1)pdm09 approximately triple that of influenza A(H3N2).

**Table 1.** Ratio of influenza A subtypes calculated based on subsets of trust data from the influenza 2023–2024 season in England. Only subtyping trusts were included. Subtyping trusts were defined as reporting at least one subtyped case in the time period

Data subset	Number of trusts	A(H1N1)pdm09 cases	A(H3N2) cases	Ratio	Lower 95% confidence interval	Upper 95% confidence interval
All reporting trusts data	28	119	38	3.13	2.17	4.51
Trusts that report subtype > = 50% of weeks with submitted data	18	96	28	3.43	2.25	5.23
Trusts that report subtype in > = 80% of weeks with submitted data	13	77	27	2.85	1.84	4.42

Finally, we investigated whether the proportion of individual cases reported with influenza subtype varied between age groups. Across individual cases, the age groups with the highest and lowest proportions of subtype reporting were the 5–14 and <1 year age groups, respectively (Figure 1c). If influenza cases in certain age groups are more likely to be subtyped (e.g., critical care cases who may be younger) and if certain age groups are more susceptible to specific influenza subtypes this could bias ratio estimates, making estimates less representative of the whole population. A chi-squared test found no significant difference in the proportion of influenza A cases reported with subtype across age groups (X_14_ = 11.284, *p* = 0.666).

Estimating the ratios of influenza subtypes circulating in a population has short- and long-term implications. In the short term, it informs the prescription of antivirals to patients since subtyping information for an individual patient’s infection may not be immediately available [3]. In the long term, it provides epidemiological context for the recommendations on the strain composition of influenza vaccines [4]. Furthermore, in the absence of widespread use of specific assays, the detection of unsubtypeable viruses supports the early recognition of emerging influenza viruses, such as influenza A(H5) subtype, which has been related to several zoonotic infections [9]. This capability is particularly important prior to the widespread introduction of specific assays, which might be prompted by such surveillance information.

The accuracy of subtype ratio estimates depends on the availability of influenza A subtype information and representativeness of the surveillance data. We identified low levels of subtype reporting across all age groups by NHS trusts, which likely limits the potential for early recognition of new influenza strains and the informing of the prescription of antivirals in seasonal and epidemic influenza outbreaks. Subtype reporting varied across regions, with the highest proportion in the East of England and Yorkshire and Humber regions. The distribution of subtype reporting trusts across NHS trust types was also highly variable, but less clear cut due to large differences in the numbers of each trust type. Despite large variation in the regularity of subtype reporting by NHS trusts, estimates of the influenza A subtype ratios using differing subsets of data drew the same overarching conclusions, that influenza A(H1N1)pdm09 was the dominant strain with approximately three times the number of cases as influenza A(H3N2). We acknowledge that these are approximate subtype ratio estimates using data from a single season. Furthermore, although the coverage of our data is relatively high, our data does not include all acute NHS trusts in England.

When there are low levels of subtype reporting, we recommend considering the factors investigated here to assess the representativeness of influenza subtype data. This is because if influenza cases in certain regions, trust types or age groups are more likely to be subtyped (e.g., critical care cases who may be younger) this could bias ratio estimates, making subtype ratio estimates less representative of the whole population.

## Data Availability

Analysis is based on the SARI Watch surveillance data, which are publicly available (Weekly_Influenza_and_COVID19_report_data_w21data.ods). The data are subject to retrospective updates and therefore is subject to small changes.
